# Potential Impacts of Climate and Land Use Change on the Water Quality of Ganga River around the Industrialized Kanpur Region

**DOI:** 10.1038/s41598-020-66171-x

**Published:** 2020-06-04

**Authors:** Sneha Santy, Pradeep Mujumdar, Govindasamy Bala

**Affiliations:** 1 Interdisciplinary Centre for Water Research, Indian Institute of Science, Bangalore, India; 2 Civil Engineering, Indian Institute of Science, Bangalore, India; 3 Centre for Atmospheric and Oceanic Sciences, Indian Institute of Science, Bangalore, India

**Keywords:** Environmental impact, Hydrology

## Abstract

The heavily industrialised Kanpur region is the most polluted stretch of the Ganga river because of excessive pollutant discharge from the industries. Agricultural runoff along with climate change further adds to the pollution risk in this industrialised stretch of Ganga. In this paper, we analyse the potential impacts of climate change and land use change on the water quality in this stretch under hypothetical scenarios using the water quality model, QUAL2K. Water quality indicators of Dissolved Oxygen (DO), Biochemical Oxygen Demand, ammonia, nitrate, total nitrogen, organic-, inorganic- and total phosphorous and faecal coliform are assessed for eight climate change and six land use land cover scenarios. Eutrophic conditions are observed in this stretch of the river for all scenarios, implying severe impacts on aquatic life. DO is identified as the most sensitive indicator to the climate change scenarios considered, while nutrients and faecal coliform are more sensitive to the land use scenarios. Increase in agricultural land area leads to larger nutrient concentration while increase in built-up area causes an increase in faecal coliform concentration. Results from this hypothetical study could provide valuable guidance for improving the water quality of the Ganges in future climate change and land use change scenarios.

## Introduction

Ganga river pollution is one of the most discussed topics on river water quality in the past few years. The uncontrolled discharge of domestic sewage without treatment, excessive pollutant discharge from the industries, agricultural runoff, etc have made the river highly polluted^1^. Ganga Action Plan (GAP) was launched in 1986 with an objective of restoring water quality to ‘Bathing class’^2^. Under GAP, several sewage treatment plants are constructed, common effluent treatment plants are constructed in places where more industries are situated, and centralized monitoring systems are made compulsory for individual industries. While these actions have contributed to improve the water quality, it is still a long way before the quality can be restored to the bathing class standards^2^. As per year 2011 records, out of the 764 grossly polluting industries discharging into Ganga river, 487 industries are from the Kanpur region^3^. Therefore, the industrialised stretch of Ganga river immediately upstream and downstream of Kanpur city can be considered as the most polluted stretch of the Ganga river. The major industries contributing to pollution of the Ganga river are tannery, sugar & distillery, pulp and paper mills^3^. Biochemical Oxygen Demand (BOD), Chemical Oxygen Demand (COD), solids, Total nitrogen (TN), Chromium, sulphide, sulphate and chloride are the major pollutants from these industries^4–7^. In addition, a significant portion of the catchment area of the river comprises of agricultural land and hence nutrient pollution (nitrogen (N) and phosphorous (P)) also becomes important^8^. The effluent disposal standards are kept constant throughout the year. Thus, the quality of the river water may get deteriorated during low flow periods even though industries are disposing their effluents at the prescribed safe limits^9^. Due to climate change, it is likely that low flows may get reduced further making the water quality worse. In addition, increasing temperatures may have varying effects on water quality indicators.

In recent studies, quantification of non-point sources (NPS), mainly due to agricultural runoff has been performed using flux method^10^, mean concentration method^11^ and SWAT model^12–14^. The export coefficient method^15^ which gives non-point source pollution from all land cover types is adopted here for representing NPS in this study. Recent studies indicate that socio-economic factors such as population, urbanisation and sewage treatment have a major role in water quality compared to the climatic factors^16^. Reduction in low flow and an increase in high flow, together with increase in water temperature are projected for the future time period (2071–2100) on a global scale^17^. Flow rate changes have been found to have more impact than stream temperature changes on water quality in streams^18^. DO is found to decrease for hypothetical climate change scenarios of increasing air temperature and decreasing streamflow in a study on Tunga Bhadra river using the water quality simulation model QUAL2K^19^.

Land use Land cover (LULC) and water quality relationship have been also analysed using Pearson regression analysis and multiple regression analysis in several studies^20–23^. These studies show that forest area has the lowest nitrate discharge, while agricultural lands have the largest^24^. Large urban areas are associated with large increases in BOD, COD and Total Suspended Solids (TSS), and higher value of BOD and COD in the dry season^25^. This has been confirmed in a study on the U-tapao river, Thailand^26^. Nitrogen loading could increase for combined scenarios of climate change and land use for late winter, while the response could be mixed for summer and spring where both impacts are in the opposite direction^27^. Combined impact studies on groundwater also reveal a greater pollution risk in future^28^. Water quality assessment of Ganga river using different water quality indices^29–31^ shows a poor water quality in many places. Recently, several studies have assessed the impact of climate change and socio-economic change on nitrogen and phosphorus flux in the Ganga basin using a process based Integrated catchment (INCA) model^32–37^. These studies find that the concentrations of nitrate, ammonia and phosphorus would decrease with increase in flow predicted for future SRES A1B climate change scenario. Socio-economic factors, sewage treatment plant capacity and effluent water quality are also found to have large impact on water quality.

Thus, past studies have shown that climate change and LULC can affect water quality substantially and the sensitivity is highly catchment dependent. The objective of our study is to quantify the individual contribution of climatic and land use parameters on water quality in the highly industrialised region of Ganga river. We also analyse the water quality for the combined climate change and land use land cover scenarios. A water quality simulation model, QUAL2K, is used for our investigations. For the quantification of non-point source pollution, we use the export coefficient method as it can be used to quantify pollution from all land use types. The water quality for the 7-day low flow with a return period of 10 years (7Q10) is the base condition for all analysis. The water quality in this base case 7Q10 will be compared to other scenarios in this study. Eight hypothetical scenarios of changes in air temperature and streamflow are constructed. A simple linear regression model is used to estimate stream temperature from air temperature corresponding to each of the scenarios. Change in water quality indicators - DO, BOD, ammonia, nitrate, total nitrogen, organic phosphorus, inorganic phosphorus, total phosphorus and faecal coliform (FC) - relative to the base condition is evaluated for each scenario. For the analysis of sensitivity to land use, historical LULC change in the catchment area contributing to the reach is considered. For the assessment of the impact of land-use change, six hypothetical land-use scenarios are considered. Only direct conversion of one land cover to another is considered to study the individual impacts. Impacts on water quality is assessed by estimating percentage change of water quality for all scenarios. For combined impact studies, water quality is analysed by changing both climatic and land use parameters.

The novelty of the work lies in modelling the combined effects of agricultural pollution, industrial pollution and climate change on water quality in a small, but representative, stretch of a highly industrialised region of the Ganga basin. The inferences drawn from such modelling studies would be immensely helpful for policy makers in identifying hotspots for making corrective interventions.

## Methods

### Study area

Ganga river is the largest river of India with a catchment area of 8,61,404 sq. km. River Bhagirathi and Alaknanda join at Devprayag to form the Ganga river. The total length of the river is 2525 km. It flows through 5 Indian states, namely, Uttarakhand, Uttar Pradesh, Bihar, Jharkhand and West Bengal. The main tributaries of the river are Yamuna, Ramganga, Gomti, Ghaghara, Gandak, Damodar, Kosi and Kali-East. The river supports a population of approximately 500 million people, providing water for sustaining livelihoods and irrigation of crops. Ganga river is also home for a large number of species of flora-fauna. About 2400 MW of hydropower is generated from it.

**Figure 1:**
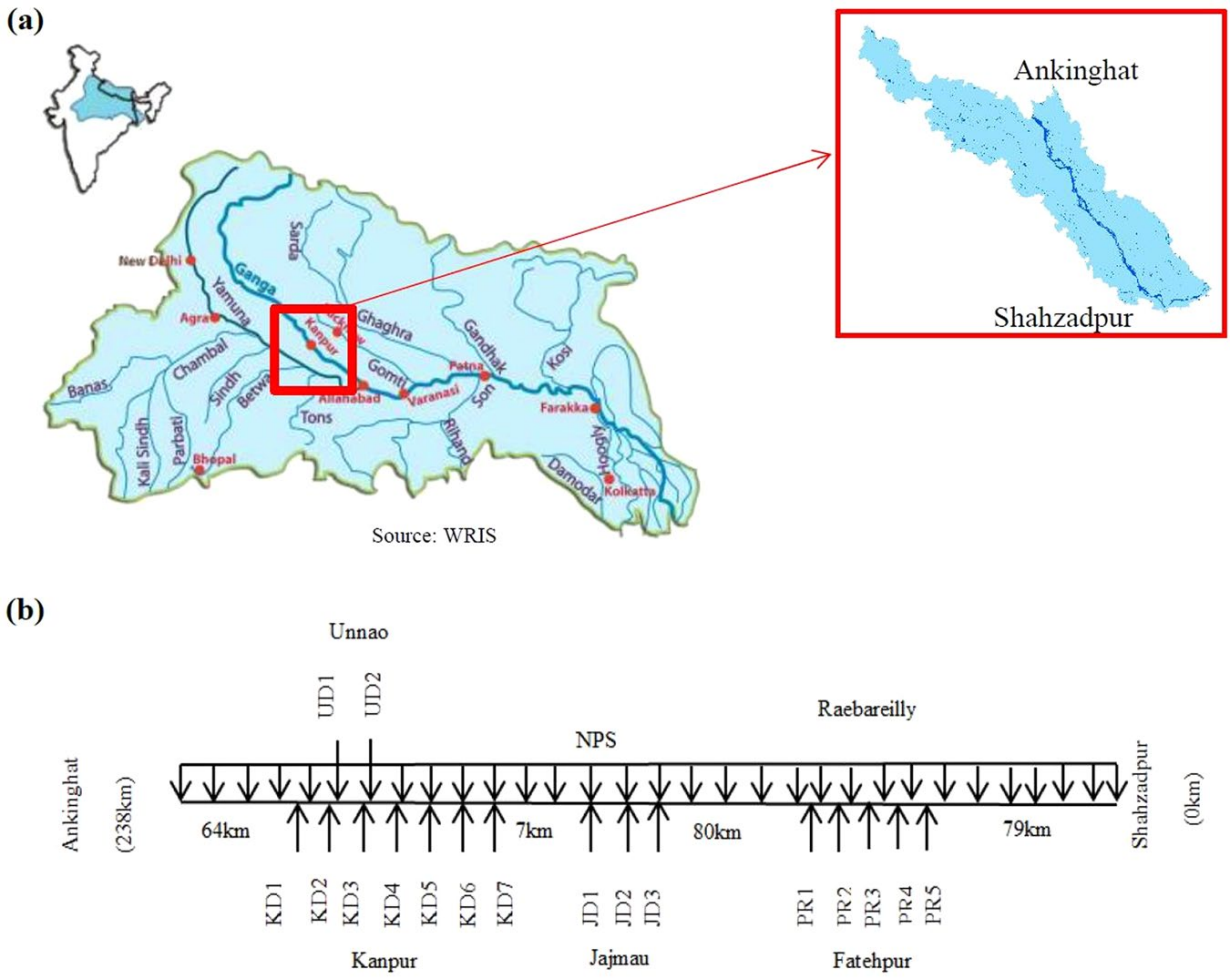
(**a**) Ganga basin with study area Ankinghat to Shahzadpur highlighted (**b**) Schematic diagram of the study area (KD: Kanpur drain; UD: Unnao drain; JD: Jajmau drain; PR: Pandu river; NPS: non-point source pollution).

The river stretch considered for this study has a length of 238 km (Fig. 1(a)). It is divided into two reaches based on the differing hydraulic characteristics: Ankinghat - Kanpur and Kanpur- Shahzadpur. The schematic diagram of the study area is shown in Fig. 1(b) and the drain data is given in Table S1.The major drains joining river at Kanpur are Ranighat drain (KD1), Sisamau nala (KD2), Bhagwatdas nala (KD3), Golaghat nala (KD4), Satti chaura (KD5) and Permiya drains (KD6). They carry waste water with high ammonia, nitrate concentration, and are contaminated with faecal coliform^3^. The Loni drain (UD1) and City jail drain (UD2) meeting river at Unnao carry high BOD and faecal coliform. Shetla bazar (JD1), Wazidpur drain (JD2) and Bhuriyaghat drain (JD3) joining the river at Jajmau cause major pollution of BOD, ammonia, nitrate, solids, phosphorus and faecal coliform^3^. Pandu river which is a house for Panki Thermal power plant drain (PR1), ICI drain (PR2), Gandanala (PR3), COD nala (PR4) and Halwa Khanda Nala (PR5), meets the Ganga river at Raebarelli with high ammonia and faecal coliform loading^3^.

### Data

The water quality data, river cross section, manning’s n, low flow data are obtained from Central Water Commission (CWC), Lucknow. The air temperature data at 1-degree grid is obtained from India Meteorological Department (IMD). The 30 m ASTER Digital Elevation Model is obtained from United States Geological Survey (USGS). Land use land cover data of 1:250,000 for the years 2005–06, 2010–11 and 2015–16 is obtained from the National Remote Sensing Centre (NRSC), Hyderabad. Data for evaporation, dew point temperature, cloud cover, wind speed at 0.25° grid is obtained from The European Centre for Medium-Range Weather Forecasts (ECMWF) reanalysis product ERA Interim (ERAI). The pollutant load data from the drains are obtained from Central Pollution Control Board (CPCB) reports.

### Scenario construction

For investigating the potential changes in water quality in the future, eight highly hypothetical climate change scenarios and 6 LULC scenarios are considered. T0FLOW10, T0FLOW20, T1FLOW0, T1FLOW10, T1FLOW20, T2FLOW0, T2FLOW10 and T2FLOW20 are the climate change scenarios considered for the study (Table S2), where the number followed by ‘T’ indicates the °C rise in air temperature and the number followed by ‘FLOW’ indicates the percentage reduction in the hydrological variable, streamflow. The water temperature is modelled using linear regression and the inputs for each scenario are given in Table S3. Six hypothetical scenarios of LULC are formulated: (1)10WAS2AGR, (2)20WAS2AGR, (3)30WAS2AGR, (4)10WAS2BLD, (5)20WAS2BLD and (6)10WAS2FOR (Table S4), where WAS2AGR, WAS2BLD and WAS2FOR represent conversion of wasteland to agriculture, built-up and forest respectively and the numbers preceding these terms represent the percentage of wasteland getting converted.

### Water quality model calibration

The water quality simulation model QUAL2K is used in this study. A detailed description of the model, calibration and validation is given in Supplementary Text S1. The 7Q10 flow and the water quality (corresponding to 2016 low flow) at Ankinghat station is given as the head water boundary condition to the model for baseline analysis (T0FLOW0). QUAL2K model is calibrated for the 2016 low flow period by changing the rate parameters and validated for 2012–2015 low flow. The range of rate parameters is taken from the literature^38–41^ and the water quality at the downstream locations of each run is compared with the water quality station data obtained from CWC. The set of rate parameters which gives the minimum RMSE between measured and modelled water quality is chosen for setting up the model. The non-point source pollution is quantified using export coefficient method (Supplementary Text S2). The data from 2005 to 2015 is used to optimize the export coefficient value for the pollutants nitrate, ammonia, phosphorus, faecal coliform and BOD. The range of export coefficient values not available in the literature is estimated using trial and error approach. Knowing the export coefficients(kg/Ha/yr) and the area of each land use type (Ha), the pollutant load (kg/yr) is calculated.

The average of diffuse flow for Ankinghat-Kanpur and Kanpur-Shahzadpur for the low flow period of 2005–2016 is used for estimating the concentration of non-point source pollutant for the baseline analysis (T0FLOW0). The resulting concentration and flow are given as diffuse source input to QUAL2K. The climatic variables such as dew point temperature, evaporation, cloud cover and wind speed data for different station points are obtained by using the nearest gridded data available from ERA Interim Reanalysis ECMWF daily dataset. All station points use the same value for the meteorological variables. Wind speed is calculated as the resultant of U wind component and V wind component (Table S5). The reach rates calibrated for low flows can be used for 7Q10 analysis^42^ (Supplementary Text S3); therefore, all rates are kept same for water quality analysis using 7Q10. The climate change scenarios, land use land cover scenarios and combined scenarios are compared with the historical 7Q10 analysis. The water quality indicators considered are DO, BOD, ammonia, nitrate, total nitrogen, organic phosphorus, inorganic phosphorus, total phosphorus and faecal coliform. Suspended solids are not, however, considered because of lack of data at station points for calibration.

## Results and Discussion

### Historical climate and land use analysis

**Figure 2:**
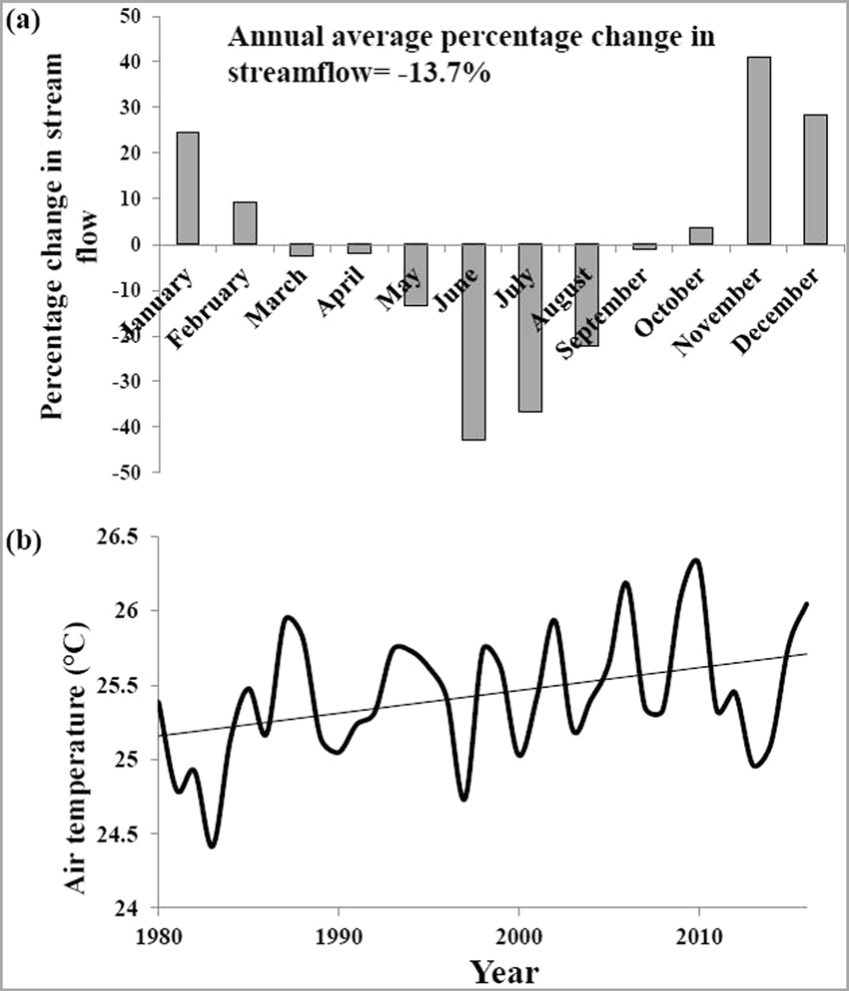
Historic analysis of (**a**) streamflow and (**b**) air temperature of Ankinghat for the period 1980–2016.

Historical water quality analysis carried out using the indicators such as minimum, mean and maximum DO, BOD, nitrate and ammonia (Fig. S1 (a),(b) and (c)) for Ankinghat, Kanpur and Shahzadpur, clearly indicates the reduction in DO and increase in BOD, nitrate and ammonia concentration for Ankinghat and Kanpur. The water quality is not found to change much for Shahzadpur. The average annual percentage change in streamflow for Ankinghat from 1968–1990 to 1991–2012 is −13.7% (Fig. 2(a)). For the Ganga basin, upstream portion consists of snow cover which can lead to increase in flow during summer season due to snow melt in a warmer climate. However, barrage constructed in the upstream of Ankinghat offsets that impact on flow at Ankinghat station, leading to reduced streamflow during summer season with warming. The air temperature plot from 1980 to 2016 (Fig. 2(b)) shows a positive trend. The average monthly temperature has increased for monsoon season and winter, with little change for summer (March and April) and decrease for May and June. The maximum increase of maximum air temperature is 1.37 °C for February and maximum increase of minimum air temperature is 1.46 °C for December (Fig. S2).

**Figure 3:**
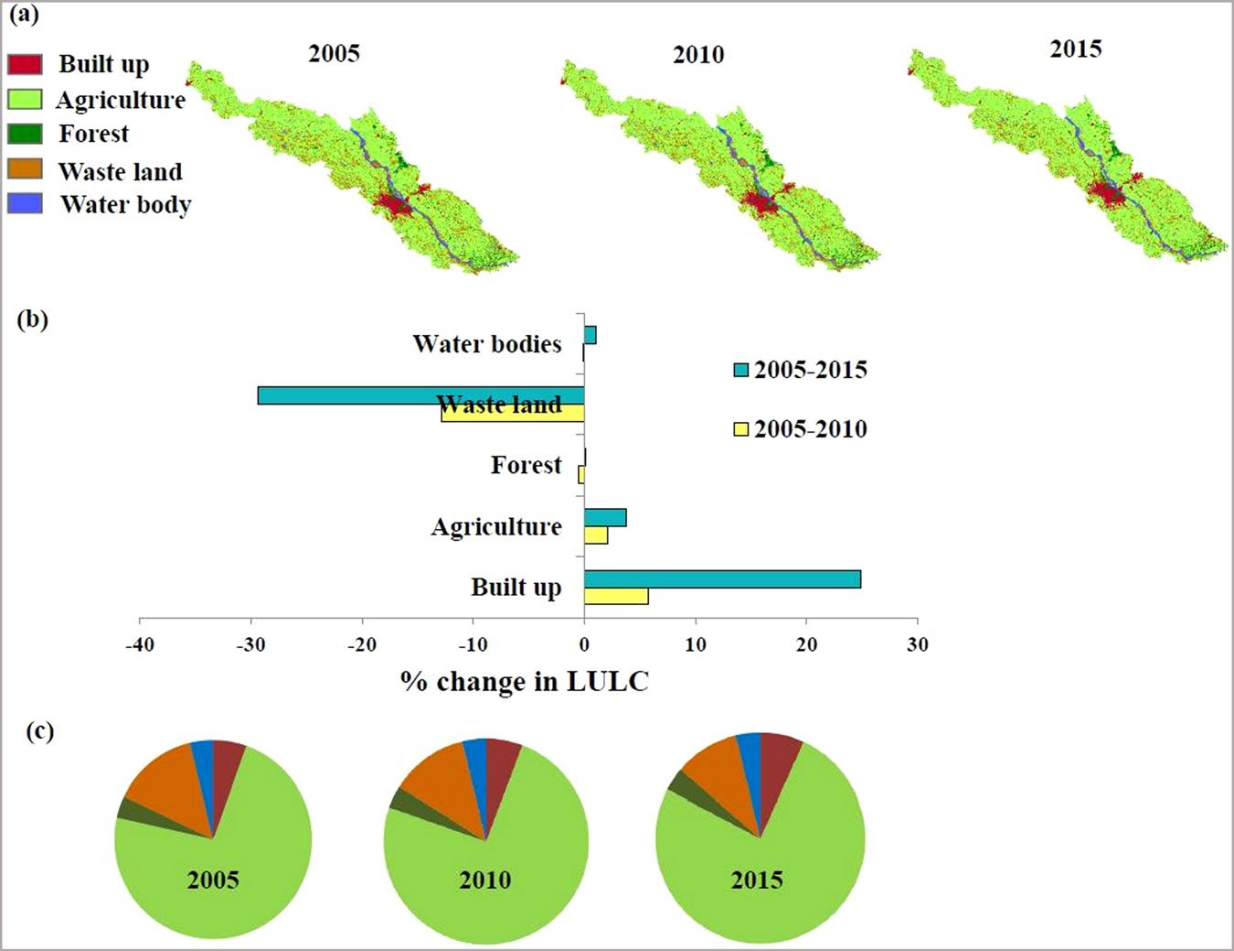
(**a**) LULC map for Ankinghat- Shahzadpur reach for 2005, 2010 and 2015 (**b**) Percentage change in each of the land use class between 2005 and 2010 and between 2005 and 2015 (**c**) Percentage distribution of each LULC class in 2005, 2010 and 2015.

Agriculture is the predominant land cover type in both the reaches Ankinghat-Kanpur and Kanpur-Shahzadpur (Fig. 3). We find that built-up area and agricultural area are increasing over the years for both the reaches with built-up area having highest percentage increase. The forest area has slightly reduced from 2005 to 2010 and slightly increased from 2010 to 2015. The waste land area has significantly reduced from 2005 to 2010 and further reduced in 2015. The water body area has slightly reduced from 2005–2015 for Ankinghat-Kanpur reach, while it slightly decreases during 2005–2010 and slightly increases during 2010–2015 for Kanpur- Shahzadpur reach. The major area land cover conversion has occurred from waste land to built-up and agricultural area. The areas for each LULC classes for both reaches are given in Table S6.

### Water quality for the design low flow (7Q10)

The export coefficient optimised for the study area is given in Supplementary Table S7. It may be noted that, significant amount of nitrate, ammonia, phosphorus, BOD and faecal coliform are exported from agricultural land and water bodies. From forest land, moderate export values are obtained for all water quality indictors considered. For the built-up area, very high export coefficient is estimated for faecal coliform and significant export coefficients for nutrients. The export coefficient values for all water quality parameters considered from waste land is minimal. The calibrated rate parameters for QUAL2K are shown in Supplementary Table S8. Other parameters are given default values. The Calibration and validation results of QUAL2K model is given in Figs. S3, S4, S5 and Table S11. R^2^ value for the validation period across all parameters is 0.6 and for individual parameters the R^2^ values are 0.7 (DO), 0.6 (BOD), 0.5 (FC), 0.6 (Nitrate) and 0.5 (TP).

**Figure 4:**
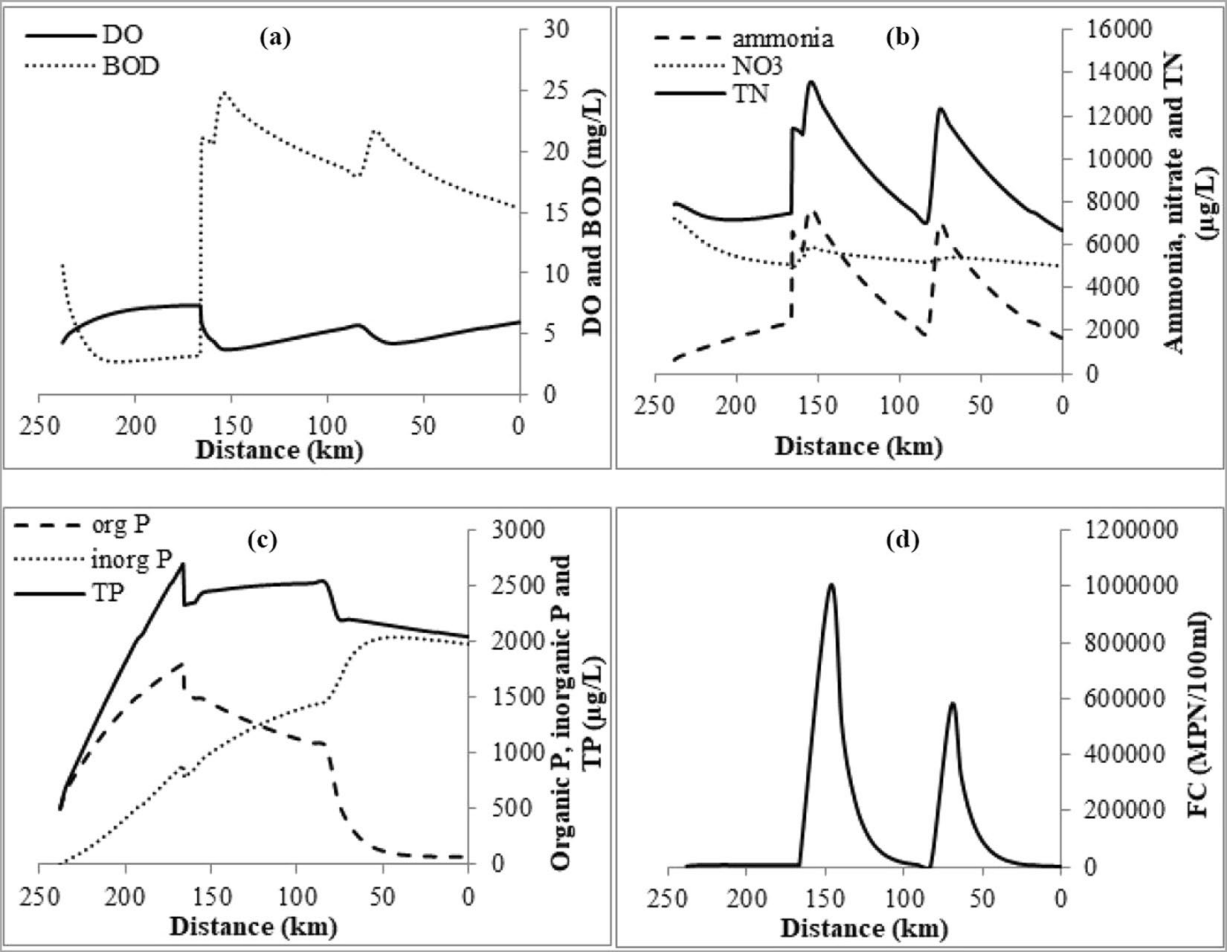
(**a**) DO & BOD profile (**b**) Ammonia, nitrate & TN profile (**c**) Organic P, inorganic P & TP profile and (**d**) FC profile along the river stretch (with Ankinghat at 238 km to Shahzadpur at 0 km) in the study area.

The starting point of DO profile (Fig. 4(a)) for this study is Ankinghat (238 km) and the zone that follows is a reaeration zone, where DO levels could increase. There is a decrease in DO corresponding to point loads at Kanpur, Jajmau and Pandu river confluence. Soon after the loading points, DO content of the river decreases because of deoxygenation. The two critical points identified are immediate downstream of Jajmau and 70 km upstream of Shahzadpur DO increases again after critical points as reaeration dominates in this region. Hence, Kanpur is in the deoxygenation zone and Shahzadpur is in the reaeration zone. BOD gets reduced from Ankinghat and suddenly increases at Kanpur due to drains carrying pollutants joining the river and again increases abruptly at the successive loading points, Jajmau and Pandu river confluence. It can be noticed that the critical DO corresponds to the maximum BOD point.

Figure 4(b) shows the mean profile of ammonia, nitrate and total nitrogen (TN) for the 7Q10 flow. Except for Unnao drains, all other drains carry significant ammonia and nitrate loading. Abrupt increases can be noted in ammonia (Fig. 4(b)) at Kanpur, Jajmau and Raebareilly where the drains join. The highest ammonia concentration simulated is 7.7 mg/L (at Jajmau), which again corresponds to the critical DO point. The plot for nitrate shows very small abrupt increases at the places where drains are joining. While comparing the values of ammonia and nitrate, we find that ammonia is getting converted to nitrate. Total nitrogen plot is similar to that of ammonia plot.

Figure 4(c) shows the profile of organic (org), inorganic (inorg) and total P (TP) for the study area. Only Jajmau drains carry P loading, so the main loading is from non-point source. The non-point loading is large in Ankinghat- Kanpur reach compared to other. In our study area, P mainly comes from fertilizers used in agricultural land. The organic P increases in first reach and slightly gets diluted when Kanpur drains joins, followed by a slight increase (not significantly noticeable) when Jajmau drains join, followed by a small dilution where Pandu river joins. Highest organic P is 1.8 mg/L. From the plot, transition of organic P to inorganic P can be noticed. Total Phosphorus value greater than 20 µg/L indicates the trophic state ‘Eutrophic’, pointing to the water body being well nourished. Profile plot (Fig. 4(c)) clearly indicates that the entire study area is eutrophic.

Figure 4(d) shows the profile of FC for the study area. It can be noted that the entire stretch is heavily polluted with FC, with major contribution from drains joining the river. FC should be 0 MPN (Most Probable Number)/100 mL for drinking water use and it should be less than 500 MPN/100 mL for bathing use. The minimum value of FC observed at Ankinghat and Shahzadpur is ~3000 MPN/100 mL, which is much higher than 500MPN/100 mL indicating that the entire stretch is not suitable for bathing purpose.

### Climate change scenarios

**Figure 5:**
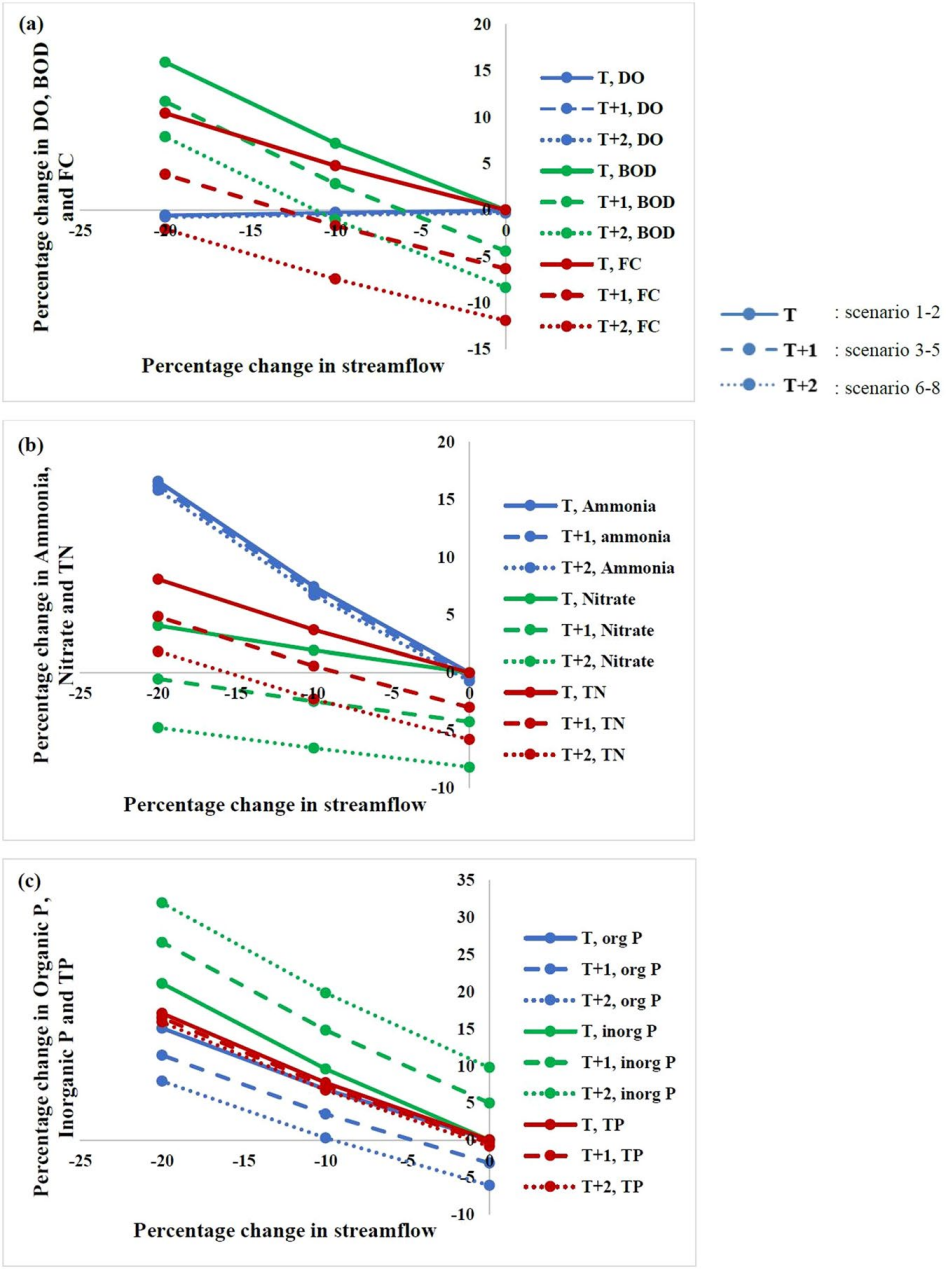
Percentage change in DO, BOD, FC, ammonia, nitrate, TN, organic P, inorganic P, and TP for climate change scenarios considered in this study for Kanpur. Blue, red and green lines indicate (**a**) DO, BOD and FC (**b**) ammonia, nitrate and TN (**c**) organic P, inorganic P and TP respectively. Solid lines, dashed line and dotted lines show scenarios 1–2, 3–5 and 6–8 respectively (Table S3).

**Figure 6:**
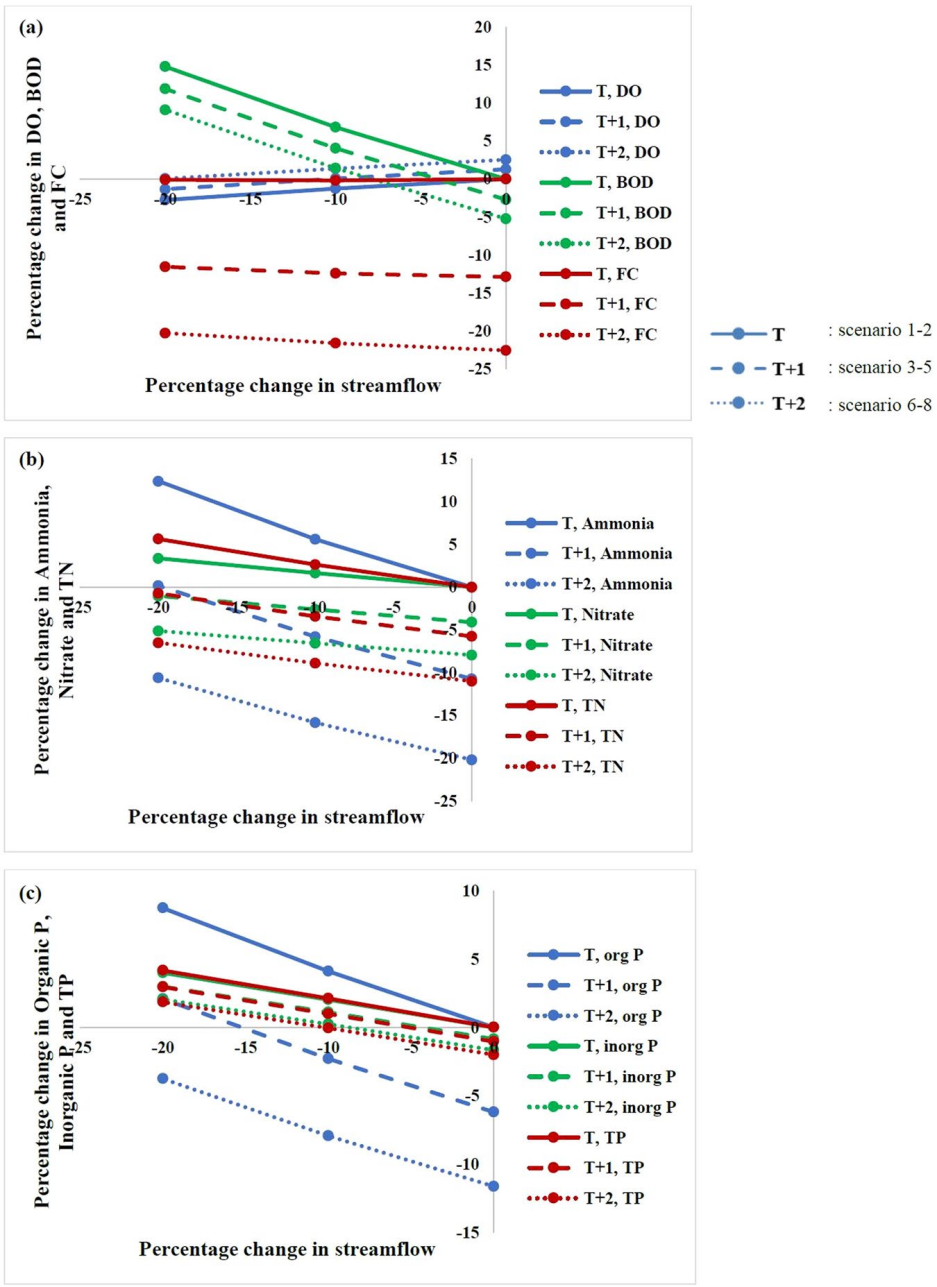
Percentage change in DO, BOD, FC, ammonia, nitrate, TN, organic P, inorganic P and TP for the hypothetical climate change scenarios considered in this study for Shahzadpur. Blue, red and green lines indicate (**a**) DO, BOD and FC (**b**) ammonia, nitrate and TN (**c**) organic P, inorganic P and TP respectively. Solid lines, dashed line and dotted lines show scenarios 1–2, 3–5 and 6–8 (Table S3) respectively.

The percentage change in water quality for the eight climate change scenarios at Kanpur and Shahzadpur is shown in Figs. 5 and 6 respectively. It is found that DO is reduced with reduction in streamflow for both the stations. This can be attributed to reduction in dilution of pollutants leading to high oxygen demand and resulting in lower DO. The points on Y-axis of the two figures also show the change with respect to temperature change alone and it clearly indicates that for Kanpur station, DO is reduced with temperature increase and for Shahzadpur station, DO increases with temperature. This is because Kanpur is in a zone where deoxygenation dominates reaeration and with increase in temperature, both the rates increases, but the net is a slight increase in deoxygenation leading to reduction of DO. Shahzadpur is in a region where reaeration dominates deoxygenation and with increase in temperature, there is a net increase in reaeration leading to an increase in DO. It may be noted that for scenarios of increased temperature and reduced streamflow, DO is very low for Kanpur while it is increased for Shahzadpur station. For the two critical points identified (Jajmau downstream and Pandu river downstream) DO is the lowest for the scenario T2FLOW20. Therefore, scenario T2FLOW20 can be considered as the critical climate change scenario for critical locations^19^ (normally downstream of pollutant loading points) and scenario T0FLOW20 for the points on reaeration zone (locations far from loading points). Maximum percentage reduction of 0.8% in DO for Kanpur is simulated for scenario T2FLOW20, and maximum percentage reduction (3%) for Shahzadpur is simulated for scenario T0FLOW20. With increase in temperature alone, rate of reaction increases and BOD is reduced. For scenarios of increased temperature and reduced streamflow, BOD is found to be increasing, which agrees with other studies^19^. The maximum percentage increase in BOD of 16% and 13%, respectively, for Kanpur and Shahzadpur is simulated for scenario T0FLOW20 where the flow is reduced by 20% but there is no temperature change. With an increase in temperature, pathogen concentration is reduced due to higher pathogen decay rate. Maximum reduction in FC for Kanpur and Shahzadpur is 12% and 23% respectively. The faecal coliform growth has been found to be reduced with increase in temperature on laboratory scale^43^ and model based studies on Lis river^44^. Tropical rivers show an increase in FC with reduction in mean annual rainfall^45^. With reduction in streamflow, concentration of FC increases and maximum increase in concentration is 10% for Kanpur for the scenario of streamflow change alone. For Shahzadpur, the percentage increase due to streamflow reduction is very small due to smaller loading and the net of combined effect is reduction in concentration.

With an increase in temperature, there is very slight reduction in ammonia at Kanpur, while the concentration is significantly reduced at Shahzadpur station (20% for T2FLOW0). For a reduction in streamflow, concentration of the pollutant increases with a maximum of 15.8% at Kanpur and 12.4% at Shahzadpur (T0FLOW20). The changes in sensitivity of ammonia in both stations is due to difference in pollution load, the load at Kanpur being approximately double that of Shahzadpur. The nitrate concentration is significantly reduced at both stations with increase in temperature. Maximum reduction in nitrate is simulated for scenario T2FLOW0 for both stations with a value of 8%. Nitrate removal efficiency is found to increase with an increase in temperature in polluted rivers^46^. With reduction in streamflow, nitrate concentration increases. But for scenario of increased temperature and reduced streamflow, net change is a reduction in nitrate with respect to base value except for scenarios T0FLOW10 &T0FLOW20. The maximum increase in nitrate concentration is simulated for scenario T0FLOW20 at both stations with maximum value of 4%. Studies on Thames river project a reduction of nitrate in 2050 due to reduced runoff and increased denitrification^47^.

With an increase in temperature, Organic Phosphorus value is depleted due to improved reaction rates with temperature. The maximum reduction in concentration is simulated for Shahzadpur and is approximately 12% for scenario T2FLOW0. With reduction in streamflow, the concentration of organic P increases up to 15% for Kanpur and 9% for Shahzadpur. The combined effects of temperature and streamflow change considered results in better water quality than the individual change of streamflow alone. The increase in concentration for streamflow reduction is large for Kanpur, because load at Kanpur is very large compared to Shahzadpur. Organic phosphorus is converted to Inorganic Phosphorus, and with an increase in temperature, the rate at which this conversion occurs increases and inorganic P concentration increases and also with reduction in streamflow the concentration again increases, leading to very large concentration in P for combined scenario at Kanpur. The maximum increase of inorganic Phosphorus modelled for Kanpur is 32% which corresponded to scenario T2FLOW20. The inorganic P load of Shahzadpur is larger than Kanpur. For Shahzadpur, with temperature increase the concentration is reduced and with streamflow reduction concentration increases and maximum concentration increase for Inorganic Phosphorus is simulated for individual scenario of streamflow change and is 4%. Maximum percentage increase in TP is for scenario T0FLOW20 and it is 17% for Kanpur and 4% for Shahzadpur. With reduction in streamflow, there is significant increase in concentration, while the temperature sensitivity is small especially at Kanpur. Phosphorus loading is not projected to change significantly in the future, when mean climate change from 8 GCMs are imposed on Michigan lake^48^. However, P is found to reduce with increasing temperature. The percentage change in each water quality parameter per °C increase in temperature and per 10% reduction in streamflow is given in Supplementary Table S9.

### Land use land cover scenarios

**Figure 7:**
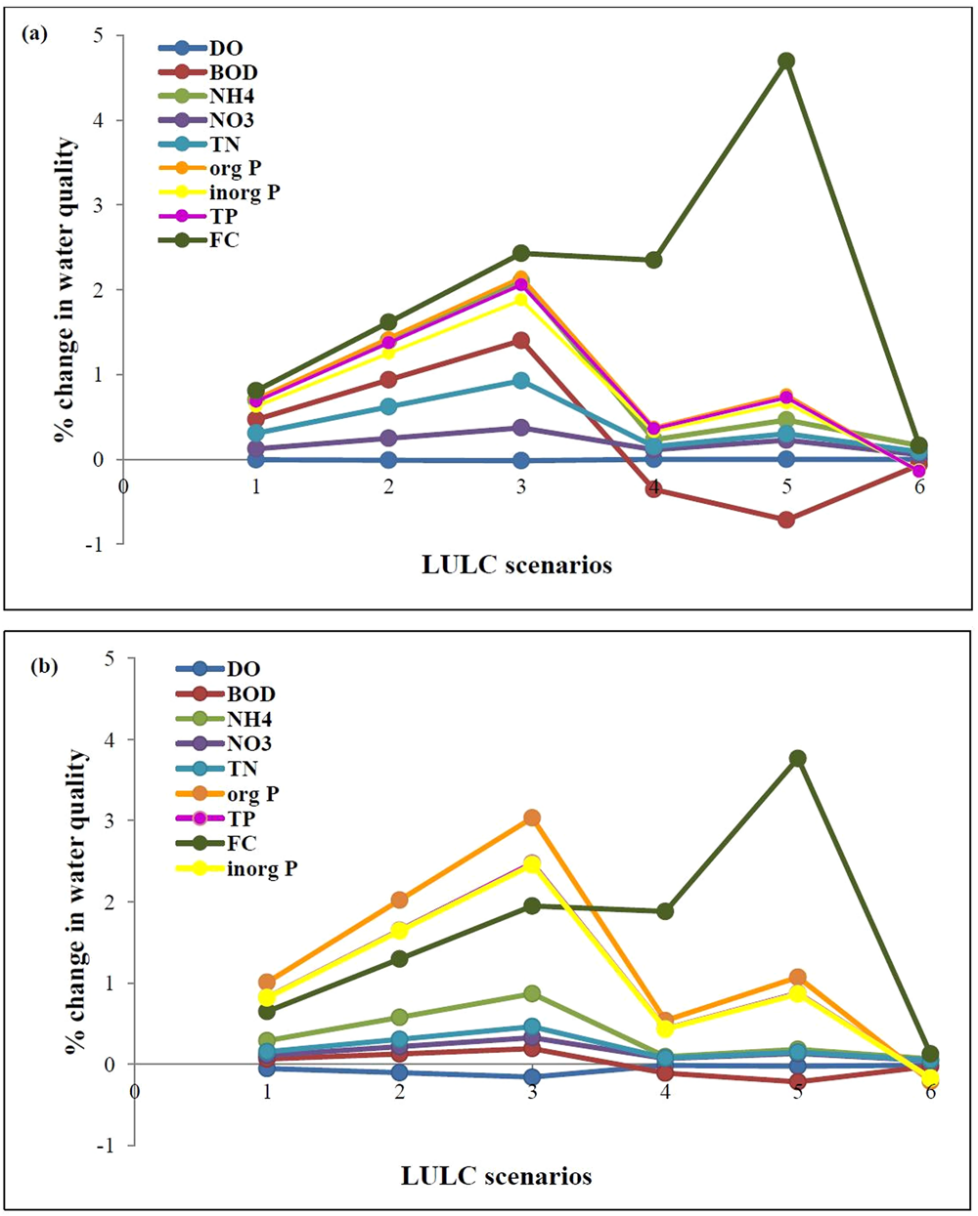
Percentage change in DO, BOD, FC, organic P, inorganic P, TP, ammonia, nitrate and TP for LULC scenarios (Table S5) for (**a**) Kanpur and (**b**) Shahzadpur.

There is hardly any change in DO for various LULC scenarios considered for Kanpur (Fig. 7). For Shahzadpur DO slightly decreases from scenario 10WAS2AGR to 30WAS2AGR, corresponding to increase in agricultural land area. This can be attributed to the corresponding increase in nutrient concentration with increase in agricultural land area. Maximum percentage reduction in DO obtained for scenario 30WAS2AGR is 0.2%. BOD is found to increase from scenario 10WAS2AGR to 30WAS2AGR, with a maximum percentage increase of 1.4% and 0.2% at Kanpur and Shahzadpur respectively. Also, there is an increase in ammonia concentration with increase in built up land area (0.5% and 0.2% for Kanpur and Shahzadpur). The least ammonia concentration is obtained for scenario 10WAS2FOR. Similar trend is noted for nitrate, TN, organic P, inorganic P and TP. Maximum percentage increase in nitrate for 30WAS2AGR at Kanpur and Shahzadpur is 0.4% and 0.3% respectively. Maximum percentage increase in TN for 30WAS2AGR at Kanpur and Shahzadpur is 0.9% and 0.5% respectively. Increase in agricultural area results in an increase of TN and nitrate concentration in a study conducted in Japan^49^. Organic P, inorganic P and TP are found to be more sensitive to LULC scenarios. Maximum percentage increase in organic P, inorganic P and TP for 30WAS2AGR is 2.1%, 1.9% and 2.1% respectively for Kanpur and 3%, 2.5% and 2.5% respectively for Shahzadpur. Also, the concentration increases for scenario 10WAS2BLD and 20WAS2BLD with maximum percentage increase of 0.7% and 0.9% at Kanpur and Shahzadpur for scenario 20WAS2BLD. For scenario 10WAS2FOR, organic P, inorganic P and TP are found to reduce, with 0.1% at Kanpur and 0.2% at Shahzadpur. For FC, concentration increases from scenario 10WAS2AGR to 30WAS2AGR with a percentage increase of 2.4% and 2% at Kanpur and Shahzadpur respectively. And there is a sharp increase for scenario 10WAS2BLD and 20WAS2BLD and hardly any increase is simulated for scenario 10WAS2FOR. The maximum percentage increase is simulated for scenario 20WAS2BLD, with an increase of 5% and 4% at Kanpur and Shahzadpur respectively. High urbanization and reduction in forestland results in increased faecal coliform^45^. Overall, the LULC analysis shows that agricultural land use leads to more degraded water quality in terms of all parameters considered. Built-up land also leads to significant pollution, mainly FC. Forest land is the one which leads to a better water quality than others. The percentage change in water quality per 10^4^Ha area LULC conversion is given in Supplementary Table S10.

### Combined climate and LULC scenarios

**Figure 8:**
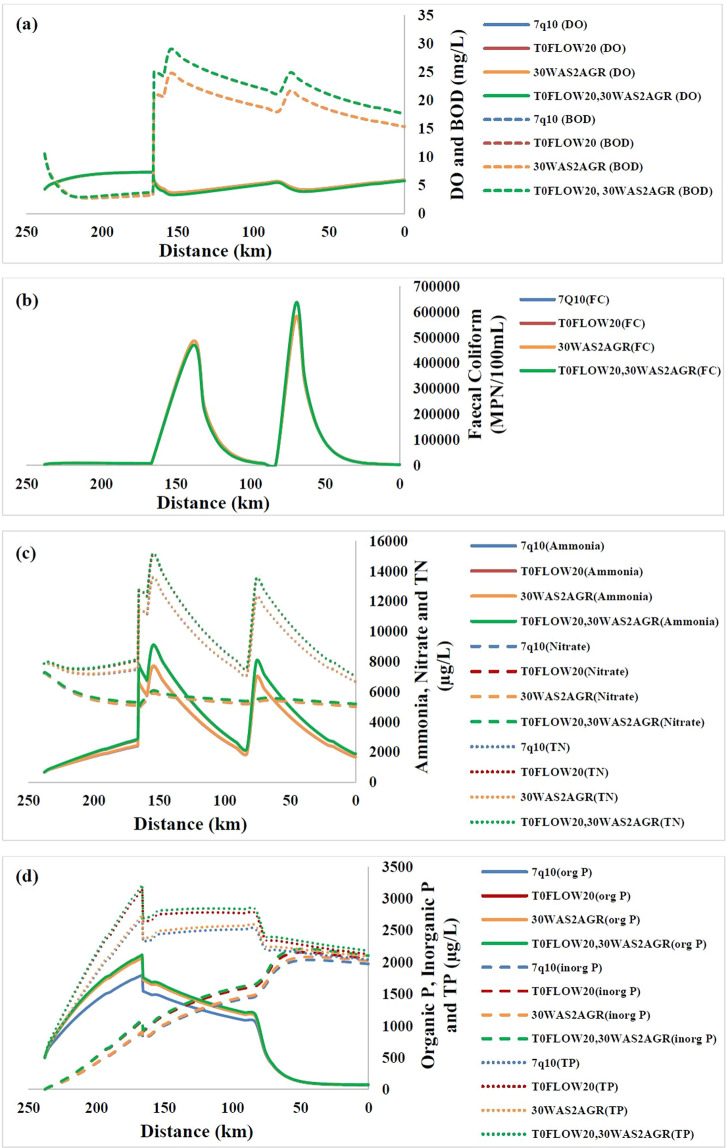
(**a**) DO and BOD profile (**b**) FC profile (**c**) Ammonia, nitrate and TN profile (**d**) Organic P, inorganic P and TP profile for combined and individual scenarios. River profile shown is from Ankinghat (238 km) to Shahzadpur (0 km).

Except for DO and Inorganic Phosphorus, the climate scenario which leads to declined water quality is the scenario T0FLOW20. Also, in terms of DO and inorganic P at Shahzadpur, least water quality is simulated for the same climate scenario, while for DO and inorganic P at Kanpur, the least water quality is simulated for the scenario T2FLOW20. Except for FC, least water quality is simulated for LULC scenario 30WAS2AGR. For faecal coliform, the worst scenario is LULC scenario 20WAS2BLD. The combined and the individual scenario are compared for 9 water quality parameters considered and one plot per parameter is shown in Fig. 8. DO of the river determines its health and its value less than 4 mg/L is not considered safe for aquatic life. DO greater than 5 mg/L is the criteria required for bathing use. DO has approximately same value up to the first loading point, after that DO varies in the order of scenarios 7Q10 > LULC > climate > combined (Fig. 8(a)). LULC scenarios do not affect DO significantly, therefore 7Q10 & LULC and climate & combined scenarios have similar values. The DO of Jajmau is below 4 mg/L even for base analysis (7Q10), which further decreases for other scenarios. The second critical point has a DO of 4.3 mg/L for base analysis with 7Q10, while for the climate and combined scenarios DO is found to drop to below 4 mg/L making it unfit for aquatic life^50^. It can be also noticed that DO decreases to less than 5 mg/L downstream of Kanpur and regains back only at 25 kms upstream of Shahzadpur, resulting in a river stretch failing to meet bathing standards in low flow periods. BOD indicates pollution in the river and BOD greater than 3 mg/L is not suitable for bathing purpose^50^. The entire stretch considered for the study has a BOD above 3 mg/L, with high BOD in the reach from Kanpur and downstream. BOD, ammonia, nitrate, TN, organic P, inorganic P, TP concentration varies in the order of scenarios, 7Q10 < LULC < climate < combined. The maximum BOD value simulated is 29 mg/L at Jajmau downstream for the combined scenario (Fig. 8(a)). Unlike other parameters, influence of climate and land use on FC shows high spatial variability (Fig. 8(b)). The first abrupt increase is downstream of Kanpur, at Unnao and Jajmau loading and second one downstream of Pandu river confluence. In the upstream reach of Kanpur, the water quality decreases progressively with scenarios of 7Q10, LULC, climate and combined effect. From downstream of Kanpur to upstream of Pandu river, water quality decreases in the order of scenarios: climate change, combined effect, 7Q10 and LULC. In the downstream of Pandu river, it varies as in the upstream of Kanpur region. Ammonia nitrogen concentration of 1200 µg/L or greater is lethal to aquatic life^50^. From Fig. 8(c) it can be noted that the limit is exceeding for the entire stretch considered. The sudden increase in ammonia corresponds to the point source inputs, with maximum ammonia concentration of 9102 µg/L at Jajmau downstream for the combined scenario.

## Conclusion

Individual impacts of climate change, and land cover change on water quality indicators such as DO, BOD, ammonia nitrogen, nitrate nitrogen, total nitrogen, organic phosphorus, inorganic phosphorus, total phosphorus and faecal coliform are analysed for the heavily industrialised stretch of the Ganga river in India. Eight highly hypothetical climate change scenarios and six LULC scenarios are used to examine the impacts, using the QUAL2K water quality model. Ganga river is found to be polluted in the heavily industrialised stretch of Kanpur for the low flow periods, because of industrial effluents and agricultural runoff. The hypothetical climate change and land use land cover scenarios considered in this study lead to higher pollution. DO of the critical points is reduced in the climate change scenarios. Except for DO, other water quality parameters are improved with temperature increase due to increased reaction kinetics at higher temperature. Streamflow reduction is a serious problem as it results in larger concentration of pollutants.

Increase in agricultural land area results in higher pollution, especially of nutrients (nitrogen and phosphorous) which can lead to eutrophication. Increase in built-up land area results in higher FC pollution. DO is, however, not found to vary much with LULC scenarios considered here. The scenario with more forestland conversion results in a better water quality. It is also found that the effects of climate change and land use change on water quality parameters add nearly linearly (Supplemental Text S4). A comparison of baseline (7Q10) with climate change scenarios, land use change scenarios and a combination of climate change and land use change shows that the water quality parameters considered here progressively deteriorate with the 7Q10, LULC, climate change and combined scenarios, except for DO which varies in reverse order. The analysis shows that entire stretch is not suitable for bathing purposes in all cases. The nutrient levels in the river shows that it is highly prone to eutrophication for all the scenarios considered. Also, the river reach downstream of Kanpur and Pandu river confluence doesn’t support aquatic life in all scenarios considered. Therefore, climate change or land use land cover change is likely to aggravate the present Ganga river pollution by industries and agricultural runoff, in the absence of any mitigative action.

Our study uses highly idealised scenarios for inferring the impacts of climate change, land use and their combined effect on water quality of Ganga river in the stretch considered. Also, the study provides an assessment of individual contribution of temperature rise and streamflow change, LULC change to agricultural, built up and forest areas on water quality, which is helpful for the policy makers and pollution control authorities to take suitable actions for a pollution free Ganga river. Projections of future climate change from GCMs and LULC will provide more realistic insight to the problem. Also, the analysis presented here, if combined with waste load allocation models^51^ will help policy makers evaluate various options to mitigate the Ganga river pollution. A better water temperature model, instead of a regression model used here is likely to give more accurate results.

Our study includes only water quality analysis during low flow period, where the water quality especially DO become critical. The Ganga Action Plan focusses on reduction in pollution load on Ganga river and hence, the industrial load and sewage load are subjected to change in the future which brings uncertainty to our water quality analysis. Also, missing values of some parameters at some stations, model and parameter uncertainty could bring in additional uncertainty to our results. Nevertheless, the qualitative findings of this study will not be altered when more realistic climate change and land use change scenarios and improved inputs to QUAL2K are considered. An investigation of the seasonality of water quality and extreme high flow events and an analysis in religious gathering areas such as Haridwar and Benaras could form the future scope of the study.

## Supplementary information

Supplementary Material.

## Data Availability

The data that support the findings of this study are available from Ministry of Water Resources, Government of India but restrictions apply to the availability of these data, which were used under license for the current study, and so are not publicly available.
