# Supplementary material for: Potential Impacts of Climate and Land Use Change on the Water Quality of Ganga River around the Industrialized Kanpur Region
Source: Sci Rep. 2020 Jun 4;10:9107. doi: 10.1038/s41598-020-66171-x (PMC7272608; doi:10.1038/s41598-020-66171-x)
Supplement: Supplementary file 1 — Supplementary Material. [file 41598_2020_66171_MOESM1_ESM.pdf]

Supplementary Material

Potential Impacts of Climate and Land Use Change on the Water Quality of Ganga River around the Industrialized Kanpur Region

SnehaSanty[1], Pradeep Mujumdar[1,2] and Govindasamy Bala[1,3]

[1]Interdisciplinary Centre for Water Research, Indian Institute of Science, Bangalore.

[2]Civil Engineering, Indian Institute of Science, Bangalore.

[3]Centre for Atmospheric and Oceanic Sciences, Indian Institute of Science, Bangalore.

Table S1: Drain data. *Flow in m^3/s; Biochemical Oxygen Demand (BOD), Ammonia nitrogen (NH3-N), Nitrate (NO3-) and Phosphorus (P) in mg/L; Faecal coliform (FC) in MPN/100ml.*

**Sl.no**	**Drain name**	**Flow**	**pH**	**BOD**	**NH_3-N**	**NO_3^-**	**FC**	**P**
Kanpur drains (KD)								
1.	Ranighat drain	0.02	7.37	173	76.2	2.02	1.6E+08	
2.	Sisamau nala	2.31	7.05	83	36.1	2.71	92000000	
3.	Bhagwatdas nala	0.2	7.24	95	48.7	2.17	92000000	
4.	Golaghat nala	0.02	7.34	143	42.9	0.876	92000000	
5.	Satti chaura	0.02	7.42	56.8	26.7	2.15	13000000	
6.	Permiya	1.75	7.16	138	52.2	2.73	92000000	
7.	Muir mill drain	0.15	7.38	85.3	40.9	2.01	1.6E+08	
Unnao drains (UD)								
1.	Loni drain	1	7.4	736			3300000	
2.	City jail drain	1.24	7.38	109			490000	
Jajmau drains (JD)								
1.	Shetla bazar	0.21	8.09	35.55	232	22.6	13000000	8.95
2.	Wazidpur drain	0.12	8.05	870	206	67.1	790000	4.45
3.	Bhuriyaghat drain	0.6	8.14	523	229	80.6	1.8	5.48
Pandu river (PR)								
1.	Panki Thermal Power Plant Drain	0.225	7.14	14	16.9	2.93	1100000	
2.	ICI Drain	2.44	8.16	42.9	193	9.85	790000	
3.	Ganda Nalla	1.4	7.17	66.6	55.2	2.87	35000000	
4.	COD Nalla	0.72	7.47	54.6	48.9	2.59	49000	
5.	HalwaKhanda Nalla	6.10	7.23	82	50.6	2	3300000	

Table S2: Climatic change scenarios considered

**Scenario no**	**Name**	**Description**
1	T0FLOW10	Air temperature unchanged & streamflow reduce by 10%
2	T0FLOW20	Air temperature unchanged & streamflow reduce by 20%
3	T1FLOW0	Air temperature increase by 1°C with no change in streamflow
4	T1FLOW10	Air temperature increase by 1°C & streamflow reduce by 10%
5	T1FLOW20	Air temperature increase by 1°C & streamflow reduce by 20%
6	T2FLOW0	Air temperature increase by 2°C with no change in streamflow
7	T2FLOW10	Air temperature increase by 2°C & streamflow reduce by 10%
8	T2FLOW20	Air temperature increase by 2°C & streamflow reduce by 20%

Table S3: Input values for each climate change scenarios

WT= 0.8523 x AT + 1.1368 (linear regression model for water temperature)

Scenario No.	Scenario names	Air temperature	Water temperature	Streamflow
1	T0FLOW10	25	22.4	30.6
2	T0FLOW20	25	22.4	27.2
3	T1FLOW0	26	23.3	34
4	T1FLOW10	26	23.3	30.6
5	T1FLOW20	26	23.3	27.2
6	T2FLOW0	27	24.1	34
7	T2FLOW10	27	24.1	30.6
8	T2FLOW20	27	24.1	27.2

Table S4: Hypothetical LULC scenarios considered in the study

**Scenario no.**	**Name**	**Description**
1	10WAS2AGR	10% wasteland converted to agricultural land
2	20WAS2AGR	20% wasteland converted to agricultural land
3	30WAS2AGR	30% wasteland converted to agricultural land
4	10WAS2BLD	10% wasteland converted to built-up land
5	20WAS2BLD	20% wasteland converted to built-up land
6	10WAS2FOR	10% wasteland converted to forest land

Table S5: Climate parameters for the reach from ERAI

**Sl.no**	**Parameter**	**Value**
1.	Evaporation (m of water equivalent)	1.57E-05
2.	Total cloud cover (%)	1.00E-10
3.	2m dew point temperature (Kelvin)	290
4.	10m U wind component (m/s)	-0.43
5.	10m V wind component (m/s)	-0.15

Table S6: Area under each LULC class for Ankinghat- Kanpur and Kanpur-Shahzadpur reach

**Reach wise area (Ha): Ankinghat- Kanpur reach**					
**Year**	**Built-up**	**Agriculture**	**Forest**	**Waste land**	**Water body**
2005	16736.97	348027.6	13799.64	57532.03	17206.01
2010	17605.19	352566.1	13789.68	52152.5	17188.73
2015	21395.5	359750.4	13844.69	41807.78	16503.86
**Reach wise area (Ha): Kanpur- Shahzadpur reach**					
**Year**	**Built-up**	**Agriculture**	**Forest**	**Waste land**	**Water body**
2005	26864.36	238614.5	15058.67	55906.16	12821.11
2010	28500.5	246295.3	14938.46	46727.16	12803.37
2015	33046.25	248999.4	15041.09	38348.01	13829.65

Table S7: Export coefficients optimized for the study area considered in this paper.

**Parameter**	**Agriculture**	**Forest**	**Built-up**	**Water body**	**Waste land**
Nitrate (kg/Ha/yr)	10	4.2	10	9.88	2
Ammonia (kg/Ha/yr)	5.8	2	2.5	7.28	0.8
Phosphorus (kg/Ha/yr)	6.9	0.5	4.4	5.3	1.6
BOD (kg/Ha/yr)	10	1	1	1.48	0.1
Faecal coliform (x10^12 MPN/Ha/yr)	1.72	0.5	4.61	3.22	0.2

Table S8: Calibrated parameters for use in QUAL2K for the study area considered in this paper.

**Sl.no**	**Parameter**	**Ankinghat-Kanpur**	**Kanpur-Shahzadpur**	**Range**
1.	Oxygen reaeration rate (d^{-1})	0.9	3.5	-
2.	Fast CBOD Oxidation rate (d^{-1})	0.02	0.8	0.02-4.2
3.	Ammonium nitrification rate (d^{-1})	0.01	1	0-10
4.	Nitrate denitrification rate (d^{-1})	2	1	0-2
5.	Sediment denitrification transfer coefficient (m/d)	1	1	0-1
6.	Organic Phosphorus hydrolysis (d^{-1})	0.2	2	0-5
7.	Inorganic Phosphorus settling velocity (m/d)	0.1	0.1	0-2
8.	Pathogen decay rate (d^{-1})	1.8	1.8	-
9.	Pathogen settling velocity (m/d)	0.1	0.1	-

Table S9: Percentage change in water quality per °C rise in temperature and per 10% reduction in streamflow.

Water quality parameter	per °C rise in temperature		per 10% reduction in streamflow	
	**Kanpur**	**Shahzadpur**	**Kanpur**	**Shahzadpur**
DO	-0.16	1.30	-0.25	-1.27
BOD	-4.42	-2.69	7.16	6.85
Ammonia	-0.37	-10.73	7.43	5.61
Nitrate	-4.26	-4.11	1.93	1.64
TN	-3.01	-5.76	3.69	2.62
Org P	-3.11	-6.19	6.82	4.09
Inorg P	4.98	-0.86	9.54	2.04
TP	-0.43	-1.03	7.73	2.11
FC	-6.32	-12.87	4.77	-0.18

Table S10: Percentage change in water quality at Kanpur and Shahzadpur when 10^4 Ha of waste land is converted to agricultural land, built-up land and forest land.

**Water quality parameter**	**Kanpur**			**Shahzadpur**		
	**Agriculture**	**Built-up**	**Forest**	**Agriculture**	**Built-up**	**Forest**
DO	-0.014	-0.004	-0.003	-1.202	-0.032	-1.168
BOD	1.117	-0.858	-0.162	-0.644	-0.271	-0.930
NH4	1.674	0.552	0.383	7.182	0.235	7.048
NO3	0.295	0.269	0.129	0.615	0.167	0.478
Org P	1.712	0.904	-0.355	2.636	1.393	-0.547
Inorg P	1.496	0.790	-0.310	2.056	1.086	-0.426
FC	1.934	5.610	0.382	1.693	4.913	0.334
TN	0.738	0.359	0.211	2.256	0.184	2.119
TP	1.640	0.866	-0.340	2.076	1.096	-0.431

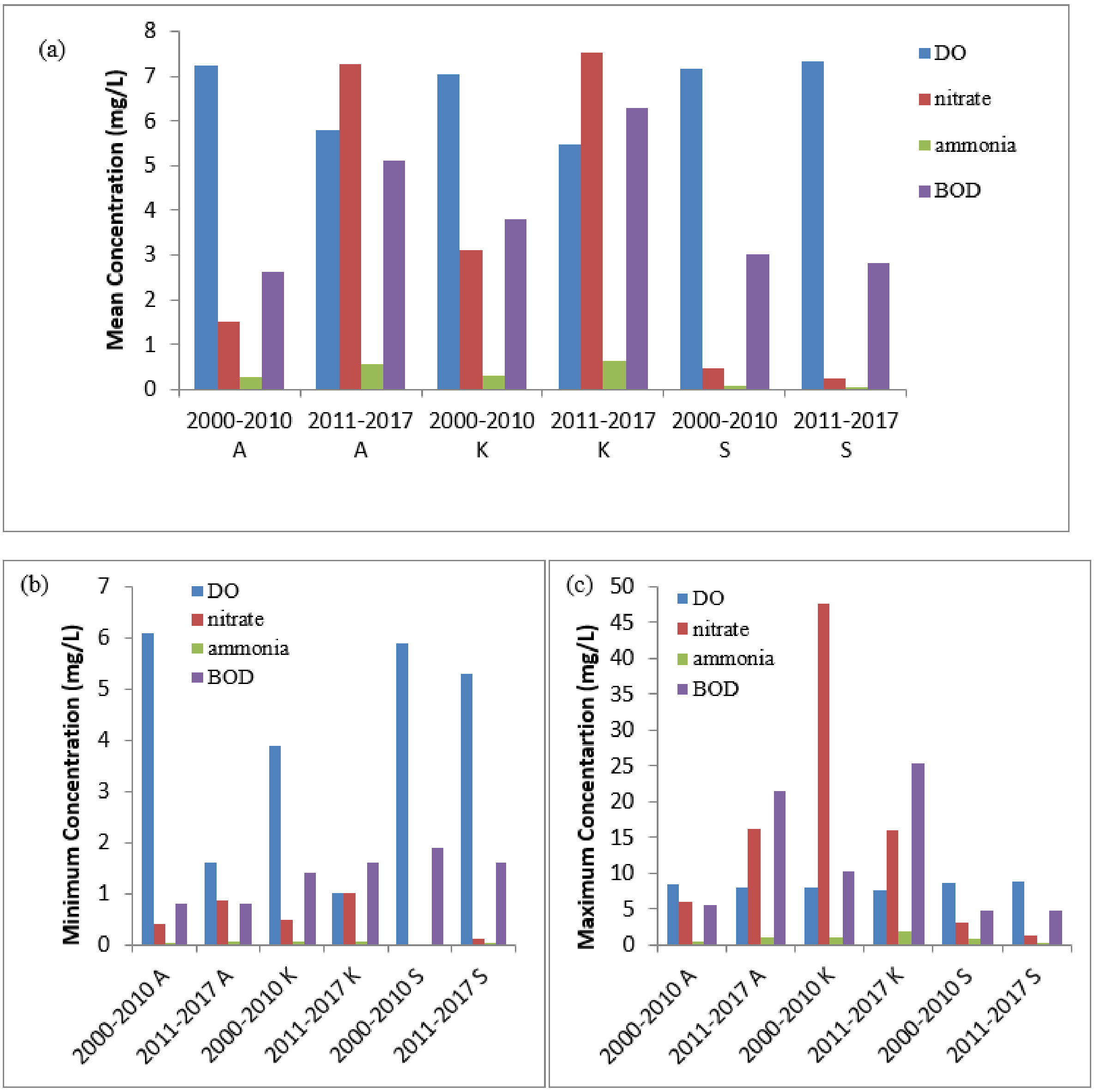

Figure S1: Comparison of (a) mean (b) minimum (c) maximum water quality at Ankinghat (A), Kanpur (K) and Shahzadpur (S) between 2000-2010 and 2011-2017 periods

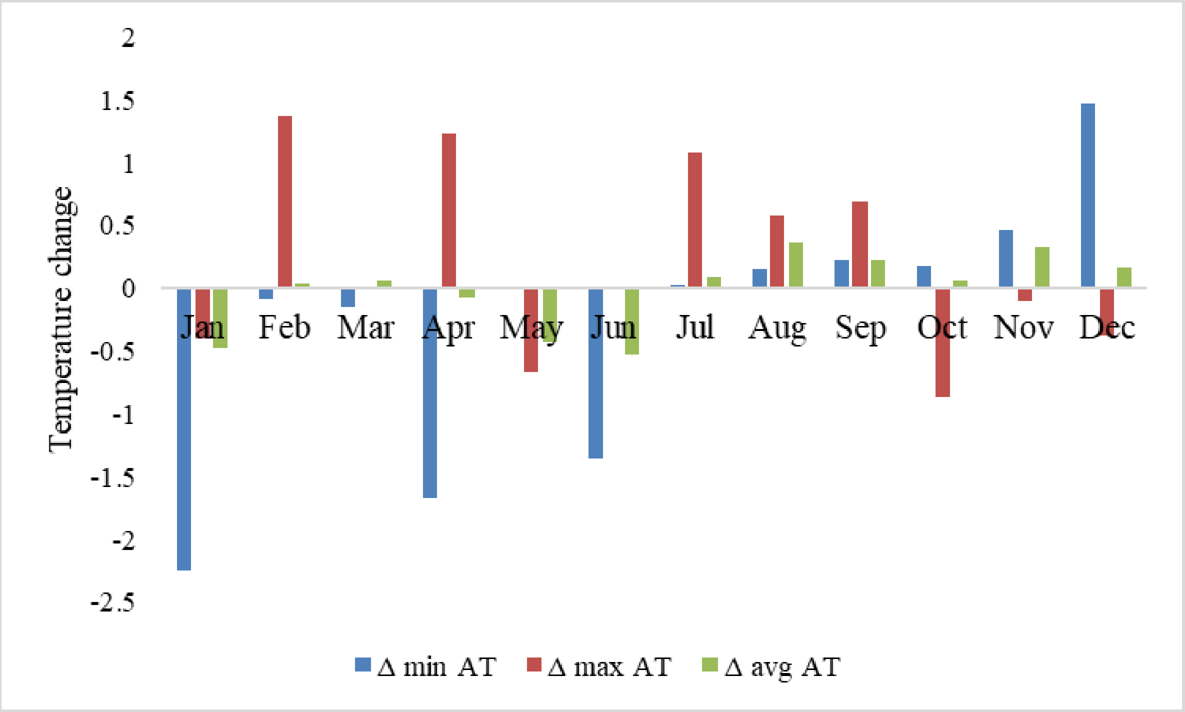

Figure S2: Maximum (red), minimum (blue) and average (green) change in monthly temperature anomaly of air temperature for Ankinghat from 1950-1980 to 1981-2016.

Section S1: Water Quality Modeling – Calibration and Validation

QUAL2K is the water quality simulation model used for the study. QUAL2K is an US Environmental Protection Agency (EPA) endorsed model written by Dr. Steven Chapra. It is a steady state model applicable for dendritic rivers or lakes. The entire river is divided into different reaches; each reach has similar hydro-geometric characteristics; reaches are divided into elements. The water quality and the flow at the head water boundary, the water quality and flow of point and non-point pollution sources, hydro-geometry of the river reaches, climatic variables for the reaches and reach rates for each water quality parameters are given as inputs to the model. The mass balance of each constituent in the reach is given by

$$\frac{\mathrm{d}C_i}{\mathrm{dt}} = \frac{Q_{i-1}}{V_i} C_{i-1} - \frac{Q_i}{V_i} C_i - \frac{Q_{ab,i}}{V_i} C_i + \frac{E'_{i-1}}{V_i} (C_{i-1} - C_i) + \frac{E'_i}{V_i} (C_{i+1} - C_i) + \frac{W_i}{V_i} + S_i$$

where C_{i-1}, C_i, C_{i+1}= concentration of the constituent in reach *i-1, i, i+1*; Q_{i-1}, Q_i =flow in the reach *i-1, i*; V_i =volume of reach *i*; $Q_{ab,i}$ = flow abstraction in reach *i*; E'_{i-1} , E'_i =dispersion between reaches *i-1* & *i*, dispersion between reaches *i* & *i+1*; W_i = the external loading of the constituent to reach i [g/d or mg/d], and S_i = sources and sinks of the constituent due to reactions and mass transfer mechanisms [$g/m^3/d$ or $mg/m^3/d$].

QUAL2K is a steady state water quality model and for setting up the model low flow of 2016 year (monthly data) and the water quality corresponding to it are given as head water condition. (2016 is the latest year data and has all parameters both for station data and point load data). For calibration and validation, the lowest flow (monthly low flow) corresponding to that year and the water quality data for that month at Ankinghat station is given as the head water boundary condition to the model. The design low flow for water quality modelling is 7Q10 and hence kept as baseline. As per Chapra, water quality rate coefficients calibrated for low flow is applicable for 7Q10 flow. The model is setup for the design low flow conditions (scenario named as T0FLOW0) with head water boundary condition as 7Q10 flow value (Section S3) and 2016 (latest data available) water quality values corresponding to low flow.

While setting up the model, Ankinghat flow is given as head water boundary condition and the flow calculated at Kanpur and actual station flow data of Kanpur is compared and change in flow value is used as diffuse source (non-point source) flow for the reach. Similarly, for Kanpur- Shahzadpur reach. For calibration and validation, the change in flow corresponding to that particular year is used. While for the baseline analysis with 7Q10 flow (T0FLOW0), the

average diffuse load in Ankinghat- Kanpur and Kanpur-Shahzadpur reach calculated by considering low flow periods of 2005-2016 is used.

The water quality and flow data at three stations in the Ankinghat- Shahzadpur stretch is available for the period 2005-2016. The model is calibrated using 2016-year low flow data. It is validated using 2012-2015 low flow periods. The model is calibrated with 15 data points and validated with 39 data points. The flow and water quality data of 2005-2016 years are used for calculating non-point source pollution. The point load data (Table S1) with all water quality parameters considered are available for 2016 year only. All the analysis is carried out keeping the point loads unchanged. By Ganga Action Plan, point loads have been reduced drastically from 2011 to 2016 year (from CPCB reports). Hence, for validation only 4 nearest years to 2016 (2012- 2015) are considered. In this 2012- 2015 period, data on some water quality parameters are missing for some years. We have used only those parameters in a year for validation for which data is available in that year.

Fig S3 shows the calibration results of the model with respect to DO, BOD, FC, Nitrate and TP. Fig S4 shows the validation graph of the model for 2012-2015 years. Simulated and observed values for DO, BOD, FC, Nitrate and TP are plotted for 39 data points (combining Ankinghat, Kanpur and Shahzadpur stations). R^2 value (average across all parameters) of 0.6 is obtained for the validation. The performance of each water quality parameter (DO, BOD, FC, Nitrate and TP) in the validation period is shown in Fig S5. The simulated and observed values of each water quality parameter for Ankinghat, Kanpur and Shahzadpur is compared for 2012-2015 years. The respective R^2 values for DO, BOD, FC, Nitrate and TP are given in Table S11.

Table S11: R^2 value for validation for DO, BOD, FC, Nitrate and TP

**Parameter**	DO	BOD	FC	Nitrate	TP
**R^2**	0.7	0.6	0.5	0.6	0.5

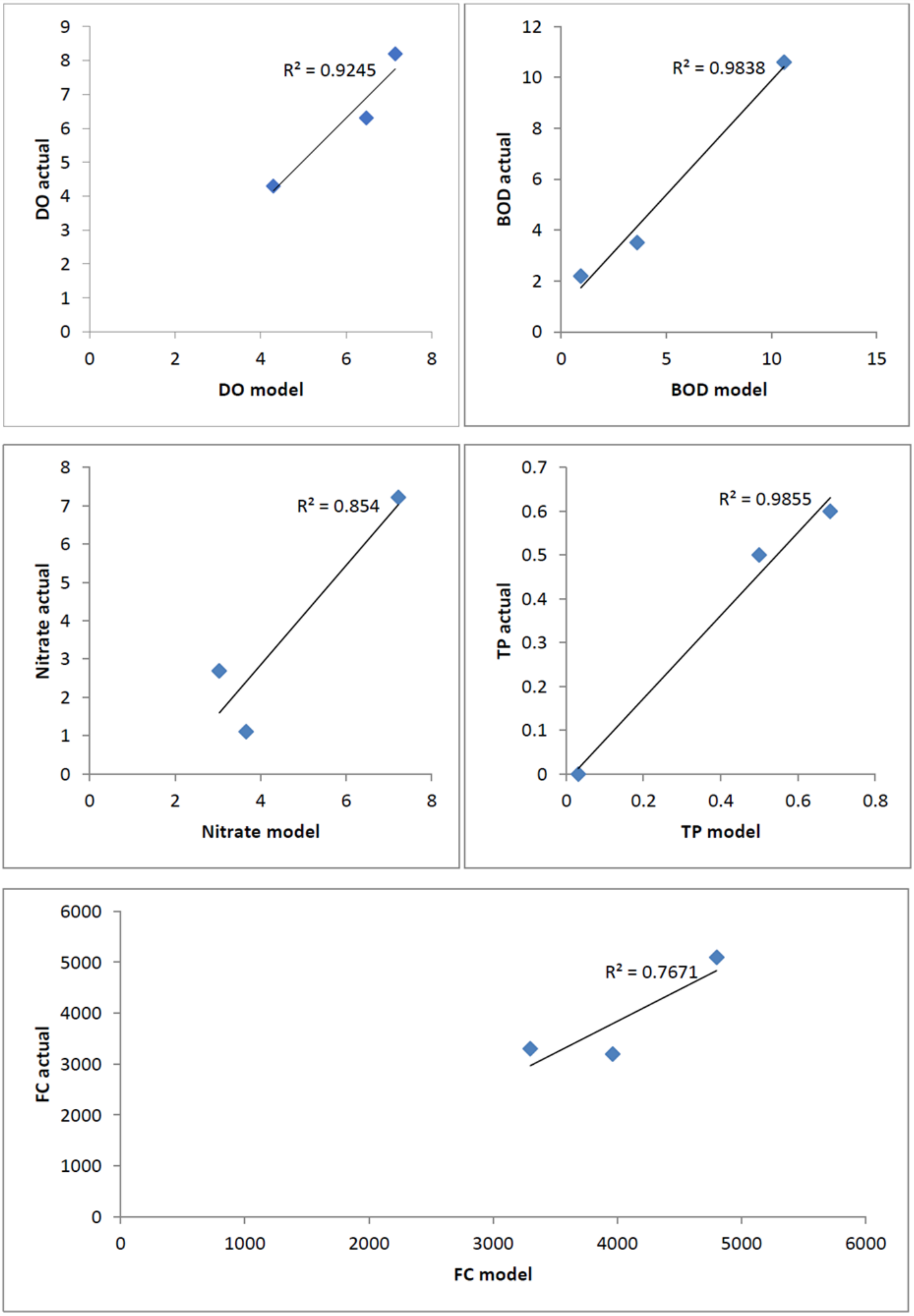

Figure S3: Model calibration using 2016 low flow with 3 station points (CWC)

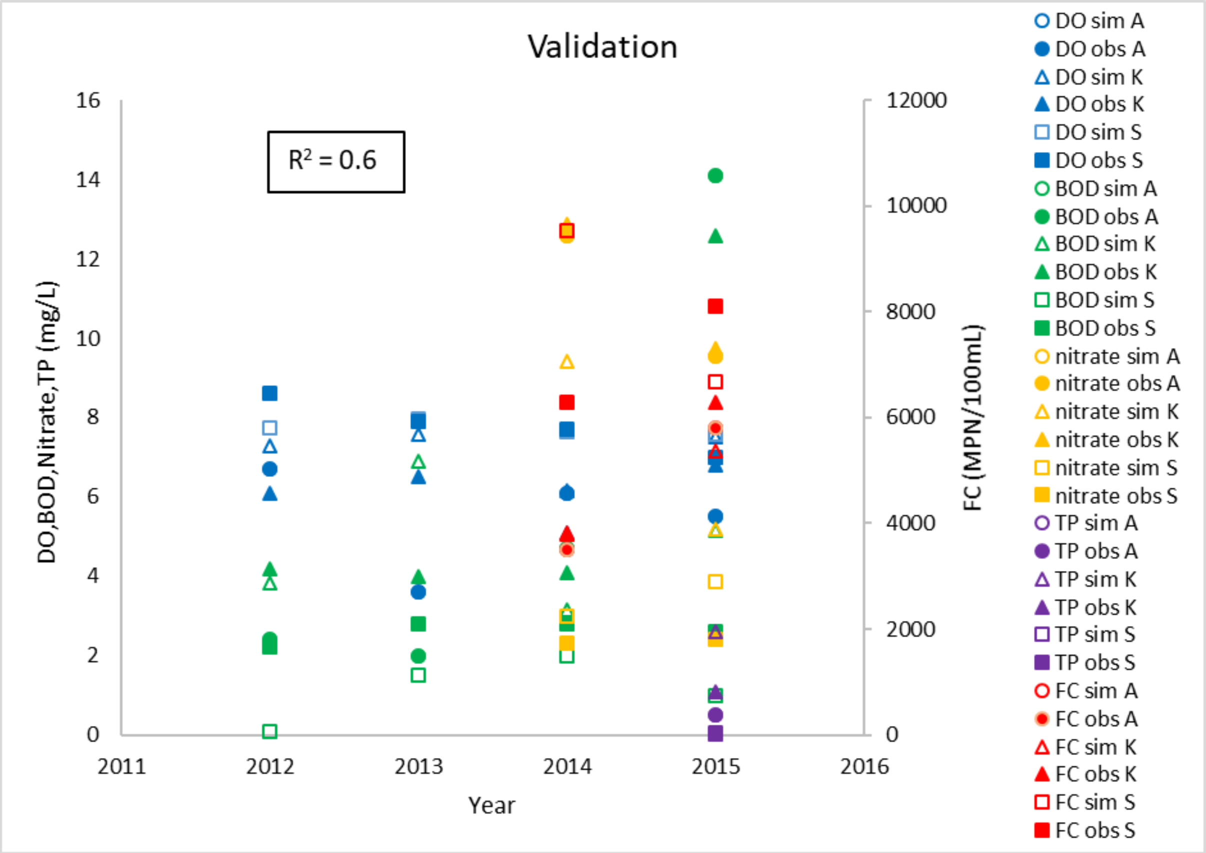

Figure S4: Validation graph of the model (with 39 data points)

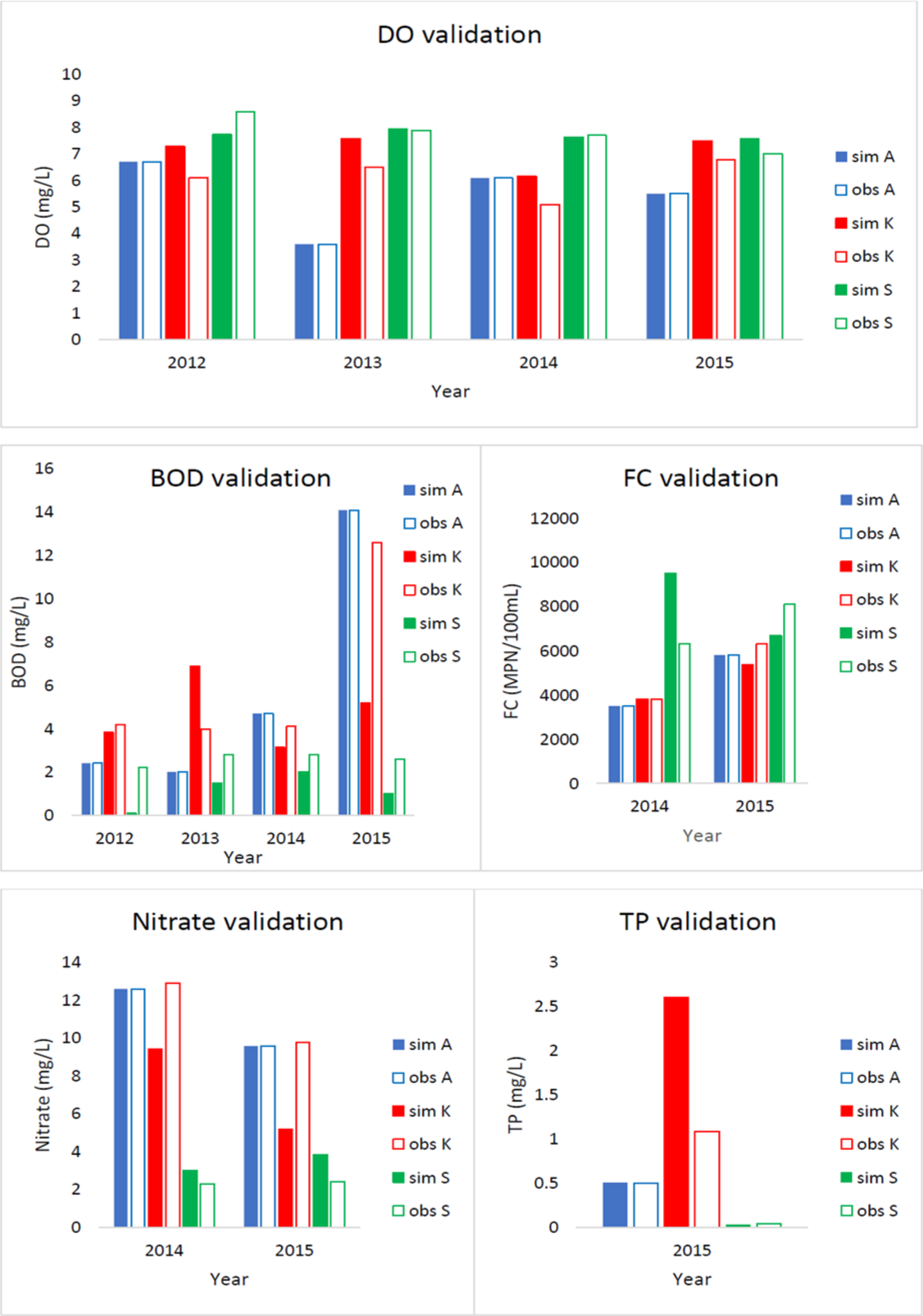

Figure S5: Validation graph of the model for DO, BOD, FC, Nitrate and TP (station wise comparison)

Section S2: Non-point source of pollution: Export coefficient method

Land Use Land Cover (LULC) data of the study area for the years 2005-06, 2010-11 and 2015-16 is obtained from NRSC, Hyderabad. We assume that LULC is linearly changing. The catchment for each of the reach is selected by subtracting the delineated catchment of downstream point of the reach from the delineated catchment of upstream point of the reach. The catchment delineation is done using ArcGIS 10.5 version. The LULC data obtained is classified with 18 land use classes, which is then grouped to 5 classes to make the computation easier. The land use classes grouped are Built-up area, Agricultural land, Forest, Wasteland and Water bodies. The range of export coefficient value for each parameter and land use is obtained from literature.

Total pollutant load in kg/yr, W=Q x C x 31536;

Q= flow rate (m^3/s); C= concentration of the pollutant (mg/L);

$W_{total}=W_{pnt}+W_{non\text{-}pnt}$

W_{total}, W_{pnt}, $W_{non\text{-}pnt}$ are total load, point load and non-point load respectively.

For example, from Fig S6,

$W_B=W_A+W_C+W_D+W_{non\text{-}pnt}$

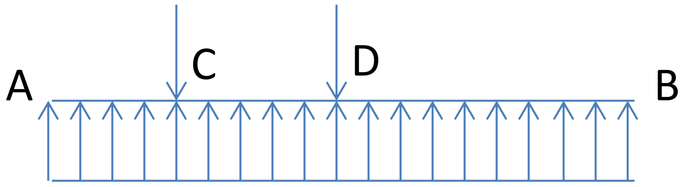

Figure S6: Intermediate river stretch with point loads at C, D and non-point load throughout.

The non-point source pollution load by export coefficient method is given by,

$W_{non\text{-}pnt}=\Sigma E_{ij} \times A_i$

E_{ij}: export coefficient of ith landuse for j^{th} parameter (kg/Ha/yr); A_i: area of ith landuse (Ha)

Section S3: 7Q10

The design low flow used for water quality problem is 7-day low flow with a return period of 10 years (7Q10). 7-day low flow corresponding to each year is calculated, sorted in order and corresponding probabilities are calculated; 7Q10 value is the flow corresponding to 10% cumulative probability. 7Q10 flow obtained for Ankinghat is 34 m^3/s. The cumulative probability of 7-day low flow is shown in the following Fig S7.

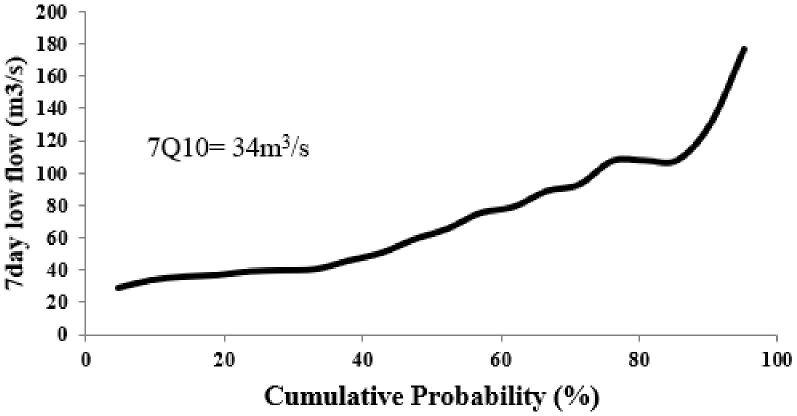

Figure S7: The cumulative probability of 7Q10 for Ankinghat

Section S4: Linearity test

In this section, we compare the percentage change in each of the parameter with the sum of change due to individual climate events (temperature change and streamflow change). Table S12 shows the comparison in terms of RMSE value. For DO and BOD, we find good agreement between combined and sum of scenarios for both the station points. For ammonia there is good agreement at Kanpur with an RMSE of 0.05, while the agreement is poor at Shahzadpur. For nitrate, the agreement is poor with high RMSE values. For TN, TP and FC, the combined effect and sum of effects are comparable at Kanpur, while it is not at Shahzadpur. For inorganic P, at Shahzadpur, there is good agreement between combined and sum of effects with RMSE of 0.4, while the RMSE has a large value of 1.4 at Kanpur. The agreement is poor for Organic P for both the stations. For some of the water quality parameters, percentage change due to the combined effect is found to be equal to sum of the percentage change due to individual events, while for others it is not. Also, it can be noted that for the same water quality parameter, no two stations show a good trend of linearity. It shows that there is significant nonlinearity and hence impact of water quality due to streamflow and temperature change cannot be assumed to add linearly. Only for water quality parameters DO and BOD there is good agreement for both the stations considered. Hence, impact of DO or BOD due to change in streamflow and temperature can be linearly added.

The combined scenarios of climate and land use are also considered for the analysis. All 8 climate change scenarios with 6 land use scenarios were checked for linearity of their effects. Table S13 shows RMSE value of comparison of actual percentage change in water quality due to combined scenarios and the sum of individual percentage change in water quality due to climate change and land use. From a comparison of 48 combined scenarios, it is found that actual and sum values show a good agreement for both the stations. Except for TP value, effects due to climate change and land use add linearly.

Table S12: RMSE (in mg/L for all parameters except FC in MPN/100ml) between the effects of climate change scenario (T1FLOW10, T1FLOW20, T2FLOW10 and T2FLOW20) and the effects of individual events of temperature change and streamflow change

	DO	BOD	ammonia	nitrate	TN	Org P	Inorg P	TP	FC
Kanpur	0.142	0.406	0.051	1.562	0.557	1.279	1.354	0.406	0.716
Shahzadpur	0.301	0.558	3.51	1.253	1.395	1.056	0.361	1.395	3.061

Table S13: RMSE between combined effects and sum of individual effects (in mg/L for all parameters except FC in MPN/100ml)

	DO	BOD	ammonia	nitrate	TN	org P	Inorg P	TP	FC
Kanpur	0.010	0.177	0.165	0.025	0.044	0.117	0.241	0.152	0.171
Shahzadpur	0.006	0.020	0.047	0.016	0.019	0.107	0.052	4.780	0.121
